# Brugada Syndrome Caused by Autonomic Dysfunction in Multiple Sclerosis

**DOI:** 10.1155/2019/3937248

**Published:** 2019-02-07

**Authors:** Michel Ibrahim, Garly Saint-Croix, Rosario Colombo

**Affiliations:** ^1^Department of Internal Medicine, Jackson Memorial Hospital/University of Miami Hospital, Miami, FL, USA; ^2^Department of Cardiovascular Medicine, Jackson Memorial Hospital, Miami, FL, USA

## Abstract

Only one case report has previously described a patient with multiple sclerosis and a type 1 Brugada pattern on the electrocardiogram. Patients with multiple sclerosis have several neurological deficits including sensory symptoms, acute or subacute motor weakness, gait disturbance, and balance problems that may lead to an increased risk of falls. Concurrent autonomic dysfunction and neurologic consequences of multiple sclerosis may precipitate both mechanical falls and falls with loss of consciousness. While mechanistically different, the type 1 Brugada pattern presents similarly with syncope due to an insufficient cardiac output during dysrhythmia. In such patients, intracardiac defibrillators have shown to prevent sudden cardiac death in patients with the Brugada syndrome. In light of these similarly presenting but unique clinical entities, MS patients who develop a syncopal event in the setting of a spontaneous type I Brugada pattern pose a diagnostic and therapeutic dilemma. This case illustrates an approach to the risks and benefits of an ICD placement in an MS patient with the type 1 Brugada pattern.

## 1. Introduction

The Brugada syndrome (BruS) is an autosomal dominant genetic disorder with variable expression characterized by typical electrocardiogram (ECG) findings with an increased risk of ventricular arrhythmias and sudden cardiac death [1]. It is essential to distinguish BruS and Brugada sign (BrS) which are two terms distinguishable by the presence or absence of symptoms. Patients with BrS are asymptomatic and have no other clinical criteria. This distinction aids in decision-making for appropriate management.

Multiple sclerosis (MS) is a chronic disease that leads to the diffuse destruction of the nervous tissue [2]. Its associations with cardiac complications have been extensively described [3]: these lesions may ultimately affect regulation of the cardiac autonomic nervous system (ANS), thus causing orthostatic signs and symptoms. MS can also present with gait instability, neuropathic pain, ataxia, and weakness, which all may increase the risk for falls.

We describe a case of a patient with multiple sclerosis presenting to the hospital after experiencing an episode of questionable syncope and ST segment elevation on the ECG that was found to have BrS.

## 2. Brugada Syndrome Diagnosis Criteria

**Figure 1:**
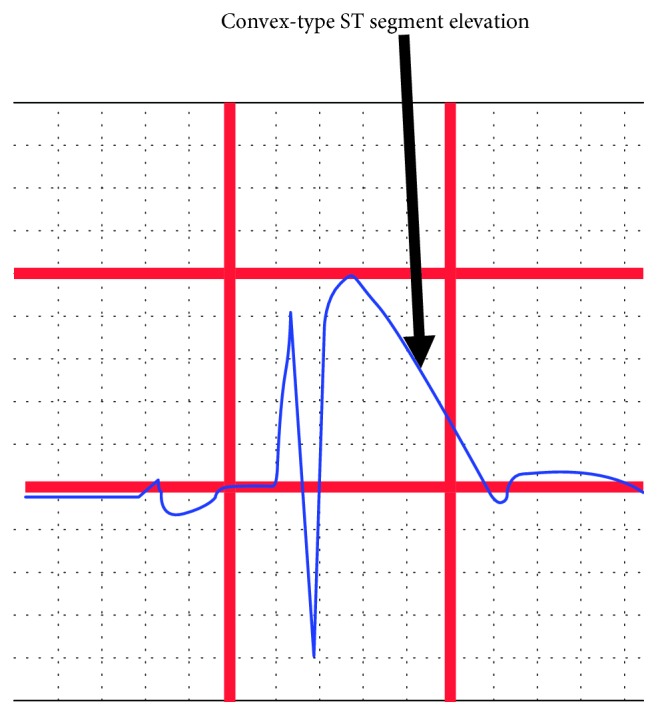
Type 1 Brugada pattern on the ECG.

BruS is diagnosed in symptomatic patients with a pseudo right bundle branch block and ST segment abnormality characteristic of type 1 morphology with a 2 mm or more elevation in the leads V1 and V2 on the ECG (Figure 1). This may occur either spontaneously or in patients with type 2 or 3 patterns during a provocative drug test with intravenous administration of class I antiarrhythmic drugs. Documented ventricular fibrillation or polymorphic VT, syncope of a probable arrhythmic cause, or a family history of sudden cardiac death [4, 5] together with a spontaneous or induced type I Brugada pattern make the diagnosis of BruS.

## 3. Autonomic Dysfunction in Multiple Sclerosis

Dysfunction of the autonomic nervous system can cause lightheadedness, dizziness, orthostatic intolerance, tachycardia or bradycardia, blood pressure fluctuations, and bowel or bladder dysfunction. In MS, cardiovascular dysfunction is mainly due to ANS dysfunction from overall demyelinating plaque burden in the medulla disrupting reflex pathways from higher cortical centers. Alternatively, there may be interference as the descending autonomic pathways course through the brainstem and spinal cord [6]. ANS dysfunction explains the orthostatic symptoms including syncope, near syncope, and orthostatic hypotension that have previously been reported in MS patients [7, 8]. Cardiovascular abnormalities may be clinical or subclinical and can also lead to sudden death in some cases [7].

## 4. Brugada Pattern and Multiple Sclerosis

In our literature search, there has been only one previously reported case of the type 1 Brugada pattern on the ECG in an asymptomatic patient with multiple sclerosis [9]. The authors stipulated that the only abnormality that could be linked to such an ECG finding is the dysfunction of the autonomic nervous system (ANS), due to lesions in the brain and spine. There is evidence that an imbalance between the sympathetic and parasympathetic nervous systems contributes significantly to the pathophysiology of the Brugada syndrome [10].

## 5. Case Presentation

**Figure 2:**
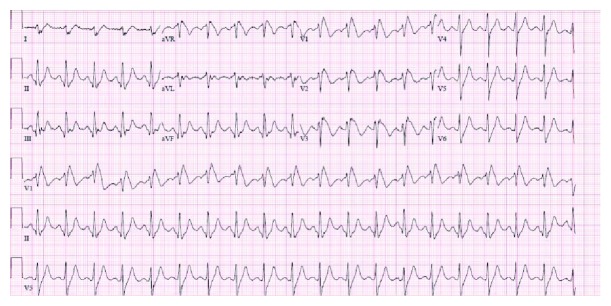
ECG: Brugada pattern. Right bundle branch block and ST segment elevation in V1 and V2.

This is a 60-year-old right-handed man with past medical history of relapsing-remitting multiple sclerosis diagnosed 20 years ago with prior beta interferon treatment for 8 years and with chronic left residual hemiparesis who presented to the emergency room after experiencing generalized weakness followed by a fall to the ground with apparent loss of consciousness. While the patient reported no loss of consciousness, he did not have memory of the events surrounding the fall. EMS was called and patient was airlifted to the nearest percutaneous intervention-capable center after the ECG showed a ST segment elevation in the leads V1 to V3, so the ST segment elevation myocardial infarction (STEMI) alert was activated. In the emergency department, the patient was without chest pain. Upon further questioning, he denied any family history of heart disease including no cardiomyopathy, heart failure, arrhythmias, or premature or sudden cardiac death. Vitals demonstrated mild tachycardia to 105 beats per minute and a temperature of 38 degrees Celsius, and labs revealed a negative troponin level. Ultimately, the ST segment elevation myocardial infarction (STEMI) alert was cancelled due to the high clinical suspicion of the type 1 Brugada pattern in a syncopal patient with anteroseptal ST elevations without chest pain. Workup for the febrile episode revealed positive serology for influenza B. Oseltamivir was started and the patient completed 5 days of treatment. The patient was no longer febrile and his tachycardia had resolved, but he continued to show a persistent type 1 Brugada pattern on the ECG during the entire hospitalization course as seen below (Figure 2). The patient subsequently went for a transthoracic echocardiogram which demonstrated a normal left and right ventricular function and no structural abnormalities. He also underwent coronary angiography, which revealed nonobstructive coronary artery disease.

Ultimately, the primary concern was to elucidate, whether the patient's initial clinical presentation represented an episode of arrhythmogenic syncope induced by the underlying Brugada syndrome, as this would lead to a recommendation for implantation of a cardiac defibrillator. Electrophysiology service was consulted and felt that the mechanism of his fall was mechanical and not related to a true syncopal event. They recommended an outpatient follow-up for consideration of an event monitor or loop recorder.

## 6. Discussion

The differential diagnosis for BrS includes several conditions that can lead to Brugada-like ECG abnormalities including acute myocardial ischemia or infarction, various central and autonomic nervous system abnormalities, and viral and febrile states among others [3, 11]. Defective myocardial sodium channels reduce sodium inflow currents, thereby reducing the duration of normal action potentials [12]. Febrile states have been reported to unmask a Brugada-like ST segment elevation secondary to reduced sodium influx at a high temperature [13]. Acute myocardial infarction or ischemia in the right ventricular outflow tract produces a ST segment elevation mimicking the Brugada syndrome probably due to the depression of I_Ca-L_ and the activation of I_K_ATP during ischemia [14]. Also, a sympathetic and parasympathetic tone imbalance may unmask the Brugada pattern on the ECG [15, 16] which may support our theory that autonomic nervous dysfunction is a possible culprit.

**Figure 3:**
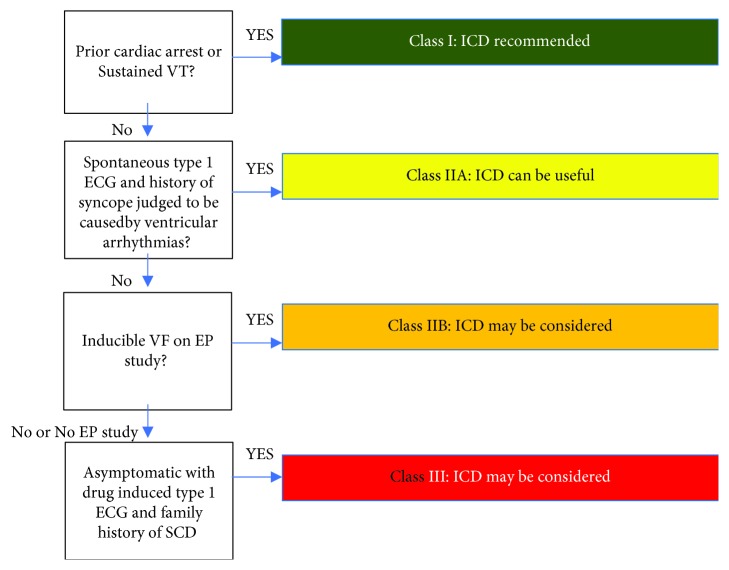
ICD in the Brugada syndrome algorithm. Consensus recommendations for ICDs in patients diagnosed with the Brugada syndrome [8].

Our patient has several different conditions that could cause him to have a ST segment elevation in V1 to V3. One of the first important ones is an acute coronary syndrome which was ruled out initially with negative serial troponin levels and definitively with a normal coronary angiogram. The patient was febrile, but he continued to have evidence of the type 1 Brugada pattern on the ECG despite improvement in his temperature and treatment for influenza. Patient's history was somewhat unclear due to loss of consciousness from arrhythmogenic syncope. Consideration can be made for an electrophysiology study; if an arrhythmia was induced, ICD implantation would be a class IIB recommendation [2]. Had the patient consciousness been secondary to an arrhythmia in the setting of the Brugada pattern on the ECG, ICD would be a class IIA recommendation [2] (Figure 3. ICD in the Brugada syndrome algorithm). Thus, it is important to be able to distinguish arrhythmogenic causes of syncope from other causes, particularly in the setting of multiple sclerosis as management and prognosis of such a patient will highly depend on this determination.

Although this patient's fever and influenza had been resolved, the classic Brugada pattern on the ECG has not been resolved. In our review, the abnormality that could be linked to the stated ECG finding is the dysfunction of the autonomic nervous system related to multiple sclerosis. Our patient did not yet have genetic testing for BruS and had no family history of sudden cardiac death. Additionally, our patient did not receive drugs with sodium channel blocking effects. In the setting of the asymptomatic Brugada syndrome, no ICD placement is recommended [17]. It is important to note that an AICD placement does not come free of risks and the patient was spared an unnecessary procedure.

Further investigation is needed in cases where the diagnostic criteria of BruS fall short, especially in patients with a typical type 1 BrS but the mechanism of syncope is in question. In our case, the patient was advised to follow closely with the electrophysiology service. But there are other modalities who have shown to be useful in such cases. In one study, the intracardiac loop recorder has shown to contribute to the exclusion of ventricular arrhythmia as a mechanism of atypical syncope in patients with electrocardiographic evidence of the type 1 Brugada pattern [18]. Continuous cardiac monitoring assists in risk stratification in patients with suspected BruS and may help to inform the possible decision for ICD implantation.

## 7. Conclusion

In summary, there is evidence that anatomical and/or functional abnormalities of the ANS can provoke ECG changes like BruS; however, the clinical significance for these patients needs further clarification. Due to the significant autonomic dysfunction that accompanies patients with multiple sclerosis, the formal diagnosis of the Brugada syndrome may prove more difficult. The prognostic significance of isolated BruS in multiple sclerosis patients remains a matter of considerable debate. In a patient like ours where the etiology of syncope is an unclear manifestation of BrS type 1, the ECG must be carefully investigated with a close follow-up, and more recently developed modalities of arrhythmia detection such as implantable loop recorders may prove to be useful towards confirming a diagnosis of the Brugada syndrome.
